# Spatio-temporal patterns of carbon, nitrogen and phosphorus in the aboveground parts of plants in alpine grasslands on the Qinghai-Xizang Plateau: Insights from a multi-faceted analysis

**DOI:** 10.1371/journal.pone.0325698

**Published:** 2025-06-13

**Authors:** Guangyu Zhang, Wei Sun, Shaowei Li, Zhiming Zhong, Gang Fu

**Affiliations:** Lhasa Plateau Ecosystem Research Station, Key Laboratory of Ecosystem Network Observation and Modeling, Institute of Geographic Sciences and Natural Resources Research, Chinese Academy of Sciences, Beijing, China; Tennessee State University, UNITED STATES OF AMERICA

## Abstract

Uncertainties in spatio-temporal patterns of nitrogen, phosphorus, especially carbon of the aboveground parts of plants limit our predictions of plant carbon sequestration capacity, nitrogen and phosphorus cycling. This study quantified the spatio-temporal patterns of carbon, nitrogen and phosphorus in the aboveground parts of plant communities in alpine grasslands of the Qinghai-Xizang Plateau in 2000–2022. The spatially averaged carbon content, nitrogen content, phosphorus content, carbon pool, nitrogen pool, phosphorus pool, carbon-nitrogen ratio, carbon-phosphorus ratio and nitrogen-phosphorus ratio were 29.96%, 1.56%, 0.32%, 28.44 g C m^-2^, 0.82 g N m^-2^, 0.07 g P m^-2^, 30.39, 271.16 and 9.59, respectively. Climate change and human activities jointly led to increases of 0.71%, 0.28%, 10.28%, 11.05%, 10.29%, 2.36%, 8.46% and 0.42% in the spatially averaged carbon content, nitrogen content, carbon pool, nitrogen pool, phosphorus pool, carbon-nitrogen ratio, carbon-phosphorus ratio and nitrogen-phosphorus ratio, while resulting in a 1.64% decrease in the phosphorus content. There were no relationships between the changes of carbon, nitrogen and phosphorus and their temporal stability. Under the influence of pure climate change, the changes of nitrogen and phosphorus pools decreased with the increase of nitrogen and phosphorus pools. Under the influence of pure human activities, the changes of nitrogen and phosphorus contents decreased with the increase of nitrogen and phosphorus contents. Therefore, the average carbon content of the aboveground parts of plant communities was < 45%, which was related to the unique climate and soil conditions of the Qinghai-Xizang Plateau. The greater the temporal stability of plant carbon, nitrogen and phosphorus, the relative change was not always smaller, which might be because the changes of plant carbon, nitrogen and phosphorus were also affected by other factors (e.g., species competition). Climate change homogenized the spatial distribution of nitrogen and phosphorus pools, and human activities homogenized the spatial distribution of nitrogen and phosphorus contents.

## 1. Introduction

Carbon, nitrogen and phosphorus, as important components of plants, play crucial roles in the energy flow and material cycling of ecosystems [1,2]. In particular, the changes in the carbon content and carbon pool of the aboveground parts of plants directly affect the carbon budget balance of terrestrial ecosystems [3,4]. Both climate change and human activities can affect the absorption, utilization, and distribution of these elements by plants, thereby changing the energy flow paths and material cycling rates of ecosystems [5]. It is of vital importance to accurately assess the absorption, storage, and release of carbon by plants for understanding the roles of ecosystems in mitigating climate change. If plants can increase carbon absorption and stabilize the carbon pool under climate change, the ecosystem will play a greater role as a carbon sink, helping to slow down the increase in the concentration of carbon dioxide in the atmosphere. Conversely, if the carbon absorption by plants decreases or the carbon pool is released more, it may exacerbate climate change. Plant nitrogen and phosphorus have significant impacts on plant carbon by affecting multiple physiological processes (e.g., photosynthesis, material metabolism, and transportation) [6,7]. Moreover, the ratio of nitrogen to phosphorus also plays an important regulatory role in this process. For example, an adequate supply of nitrogen helps plants synthesize more photosynthetic enzymes and increase the chlorophyll content, which can promote the efficient progress of photosynthesis, enhance the carbon assimilation ability of plants, and is conducive to the accumulation of organic carbon by plants. Phosphorus can promote the transportation of photosynthetic products from photosynthetic organs to other parts, which helps plants rationally distribute photosynthetic products and allocate carbon to the parts where it needs to be stored or utilized. Previous researchers have carried out a large number of studies on the spatio-temporal patterns of plant carbon, nitrogen and phosphorus, and their responses to global changes [1,8]. These studies are helpful for in-depth understanding of the dynamic balance of energy and matter in ecosystems, as well as the maintenance mechanism of ecosystem functions. They can also predict the change trends of ecosystem structures and provide a scientific basis for maintaining the stability of ecosystems. However, there are still some deficiencies in the previous studies. First, although in certain model accuracy assessment studies, the plant carbon content has been treated as a constant (e.g., 45%) [9,10], several other studies have revealed that climate change and human activities are capable of modifying the plant carbon content [11,12]. That is, there remains no unanimous conclusion regarding the specific value of the plant carbon content. Second, according to principles such as the principle of resource allocation stability, the principle of ecological balance, and the principle of plant growth adaptability, with the increase in temporal stability, the variation amplitudes of carbon, nitrogen, and phosphorus in the aboveground parts of plants would decrease [13]. By contrast, some studies have shown that the variation of a variable is not in a straightforward negative correlation with the temporal stability of that variable [14,15]. Therefore, in order to accurately quantify the carbon sequestration potential of global plants, and nitrogen and phosphorus cycling, it is necessary to accurately quantify plant carbon/nitrogen/phosphorus content/pool and their change laws under global change.

The Qinghai-Xizang Plateau, known as the “Roof of the world”, is the habitat of rare animals and plants. The alpine grassland ecosystem are unique and of great significance in the global ecological pattern. The alpine grasslands on the plateau are vast. The high altitude and low temperature lead to slow plant growth and physiological and ecological characteristics different from those in lowland areas. The dynamics of carbon and carbon pool in the aboveground parts of plants provide crucial evidence for the laws of carbon fixation and release in extreme environments, which helps to optimize the global carbon cycle model and accurately predict the impact of climate change and human activities on the global carbon balance. Its special soil texture, soil-forming process and vegetation types result in unique circulation paths and conversion efficiencies of nitrogen and phosphorus between plants, soil and atmosphere. This provides a basis for nutrient management strategies for the planning of animal husbandry on the plateau and the prevention of grassland degradation, and can prevent human activities from disrupting the nitrogen and phosphorus balance and thus affecting the stability and productivity of the ecosystem. The Qinghai-Xizang Plateau is under the dual pressures of climate change and human activities. The temperature is rising, the precipitation pattern is changing, and human activities are becoming more frequent [16]. Many studies have focused on the laws and driving mechanisms of the impacts of these two factors on the carbon, nitrogen and phosphorus in the aboveground parts of alpine grassland plants [16,17]. These studies can warn of changes in the ecosystem and provide bases for adaptive management to ensure the sustainable development of the system and the stable exertion of ecological service functions. However, there are still some uncertain factors in the relevant research that need to be further explored. First, previous studies mainly focused on the spatio-temporal pattern laws of carbon, nitrogen and phosphorus in the aboveground parts of alpine grassland plants, while relatively few studies have been conducted on their temporal stability. Secondly, there are no unanimous conclusions on whether external disturbances homogenize or heterogenize the spatial pattern of carbon, nitrogen and phosphorus in alpine grassland plants [7,18]. Therefore, to provide more scientific and effective strategies for the sustainable management and protection of this unique and fragile ecosystem in the context of global change, relevant research should be strengthened.

This study quantified the spatio-temporal patterns of carbon, nitrogen and phosphorus contents, carbon, nitrogen and phosphorus pools, and the ratios between carbon, nitrogen and phosphorus in the aboveground parts of plants from 2000 to 2022, and the spatial pattern of their temporal stabilities. The main objectives were to explore (1) the relationship between the average carbon content in the aboveground parts of alpine grassland plants on the Qinghai-Xizang Plateau and 45%; (2) the influence laws of climate change and human activities on the carbon, nitrogen and phosphorus variables in the aboveground parts of plants; (3) the relationship between the changes of plant carbon, nitrogen and phosphorus and their temporal stabilities; and (4) the influence of climate change and human activities on the spatial patterns of carbon, nitrogen and phosphorus variables in the aboveground parts of plants in alpine grasslands on the Qinghai-Xizang Plateau.

## 2. Materials and methods

### 2.1. Study area and data

The study area included all alpine grassland regions on the Qinghai-Xizang Plateau, which was mainly covered by grasslands. The mean altitude of the Qinghai-Xizang Plateau is above 4000 m. The air is thin and the atmospheric heat preservation effect on the ground is weak, resulting in relatively low temperatures. Due to the poor heat preservation effect of the atmosphere over the plateau, the solar radiation is strong during the day, and the ground heats up rapidly, leading to relatively high temperatures. However, at night, the ground cools down quickly, and the temperature drops sharply, with a daily temperature difference that can reach 20–30°C or even greater. The precipitation distribution on the Qinghai-Xizang Plateau is extremely uneven. The southeastern part is affected by the warm and humid air currents from the Indian Ocean, with relatively abundant precipitation. The annual precipitation can reach 500–1000 mm or even more, forming a relatively humid climate environment. In the northwestern region, however, due to its remoteness from the ocean, it is difficult for water vapor to reach, and the precipitation is scarce. The annual precipitation is mostly below 100 mm, and in some areas, it is even <50 mm, presenting arid and semi-arid climate characteristics. In addition, the solar radiation is intense and there are many windy days. The alpine grasslands in different regions of the Qinghai-Xizang Plateau experienced different magnitudes of climate change (e.g., climate warming, precipitation change, and radiation change), soil carbon, nitrogen and phosphorus changes, and forage nutritional quality changes. The effect of human activities on alpine grasslands cannot be ignored either.

The monthly mean temperature, total precipitation, and total radiation data in 2000–2022 were sourced from “*A high-resolution near-surface meteorological forcing dataset for the Third Pole region* (TPMFD, 1979 - 2022)” [19,20]. This dataset had higher precision compared to the current mainstream reanalysis data and can be used for climate analysis in the Third Pole region and as input for models related to land surface, hydrology, and ecology [19,20]. The monthly-scale normalized difference vegetation index (NDVI) was derived from the product data (MOD13A3, spatial resolution of 1 km × 1 km, temporal resolution of 1 month) of the Moderate Resolution Imaging Spectroradiometer (MODIS). The annual mean temperature, total precipitation, total radiation and maximum NDVI (NDVI_max_) were obtained based on the original monthly-scale temperature, precipitation, radiation and NDVI data in 2000–2022, respectively. Soil organic carbon (SOC), total nitrogen (STN), total phosphorus (STP), ammonium nitrogen (NH_4_^+^-N), nitrate nitrogen (NO_3_^-^-N), available phosphorus (SAP), pH, ratio of SOC to STN (SC:N), ratio of SOC to STP (SC:P), and ratio of STN to STP (SN:P) at 0–10 cm across the whole grasslands on the Qinghai-Xizang Plateau were obtained based on the random forest models constructed by our previous studies under the combined effect of climate change and human activities, and the single effect of climate change, respectively [21–23]. The contents of carbon, nitrogen and phosphorus, carbon, nitrogen and phosphorus pools, carbon-nitrogen ratio, carbon-phosphorus ratio and nitrogen-phosphorus ratio of the aboveground parts of plant communities in the alpine grasslands across the entire Qinghai-Xizang Plateau from 2000 to 2022 under the combined effects of climate change and human activities as well as under the sole effect of climate change were also obtained based on the constructed random forest models (not published), respectively. The random forest models of these plant variables had relative higher predicted accuracies (relative bias ranged from –2.94% to 4.77%). The mean observed plant carbon content used in the construction of the random forest models was 39.70% (22.79–49.30%) and 36.21% (11.80–52.10%) under the single effect of climate change, and the combined effects of climate change and human activities, respectively (S1 Fig in S1 File). Referring to previous studies [14,15], variables such as soil carbon, nitrogen, and phosphorus and aboveground plant carbon, nitrogen, and phosphorus variables under the single influence of human activities are the ratios of the relevant variables under the combined influence of climate change and human activities to the relevant variables under the single influence of climate change.

The Carbon_C_C+H_, Nitrogen_C_C+H_, Phosphorus_C_C+H_, Carbon_P_C+H_, Nitrogen_P_C+H_, Phosphorus_P_C+H_, C:N_C+H_, C:P_C+H_ and N:P_C+H_ indicated the carbon content, nitrogen content, phosphorus content, carbon pool, nitrogen pool, phosphorus pool, the ratio of carbon to nitrogen, the ratio of carbon to phosphorus, and the ratio of nitrogen to phosphorus of plant communities under the combined effect of climate change and human activities, respectively. Similarly, the Carbon_C_C_, Nitrogen_C_C_, Phosphorus_C_C_, Carbon_P_C_, Nitrogen_P_C_, Phosphorus_P_C_, C:N_C_, C:P_C_ and N:P_C_ indicated the concerned variables under the single effect of climate change. However, the Carbon_C_H_, Nitrogen_C_H_, Phosphorus_C_H_, Carbon_P_H_, Nitrogen_P_H_, Phosphorus_P_H_, C:N_H_, C:P_H_ and N:P_H_ indicated the concerned variables under the single effect of human activities.

### 2.2. Statistical analyses

Referring to previous studies [24,25], we calculated the temporal stability of relevant variables (i.e., the mean value of the variables from 2000 to 2022 divided by its standard deviation) of all grassland pixels. We used the sens.slope function of the trend package to calculate the rate of change (Δ) of all grassland pixels from 2000 to 2022. We obtained the relative change of the concerned variables based on the rate of change and the value in 2000 for any pixel. Based on previous studies [14], we quantified whether climate change or human activities dominated the changes in carbon, nitrogen, and phosphorus of the aboveground parts of plants. We quantified the linear regressions of carbon, nitrogen and phosphorus of the aboveground parts of plants and their rates of change, which can used to analyze whether external disturbances homogenized or heterogenized the spatial pattern of this variable [14,15]. We also quantified the relationships between the relative changes of carbon, nitrogen and phosphorus of the aboveground parts of plants and their temporal stability, which can be used to explore whether the higher the temporal stability of the variable, the lower its relative change [14,15]. In addition, we used the varpart function (vegan package) to quantify the relationships between the dependent variable and the independent variables. All statistical analyses were conducted using R software.

## 3. Results

**Fig 1:**
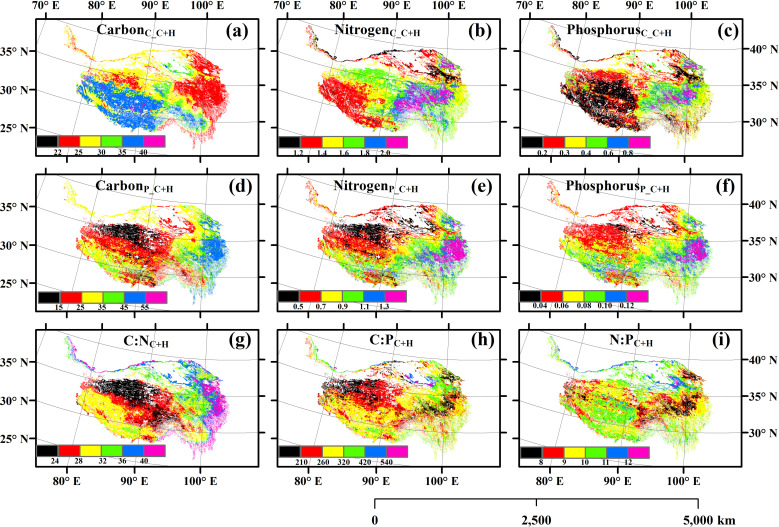
The spatial distribution of (a) carbon content, (b) nitrogen content, (c) phosphorus content, (d) carbon pool, (e) nitrogen pool, (f) phosphorus pool, (g) ratio of carbon to nitrogen (C:N), (h) ratio of carbon to phosphorus (C:P), and (i) ratio of nitrogen to phosphorus (N:P). C + H: simultaneously affected by climate change and human activities.

There were significant spatial variations in the self-, temporal stability and relative change of the carbon, nitrogen and phosphorus contents, carbon, nitrogen and phosphorus pools, and carbon, nitrogen and phosphorus ratios of the aboveground parts of plants (Figs 1^3^, S2–S7 in S1 File). Climate change homogenized the spatial distribution of nitrogen and phosphorus pools, and human activities homogenized the spatial distribution of nitrogen and phosphorus contents (S8–S9 Figs in S1 File). However, the impact of their interactive effects on the spatial homogenization of these variables was relatively minor (S10 Fig in S1 File).

**Fig 2:**
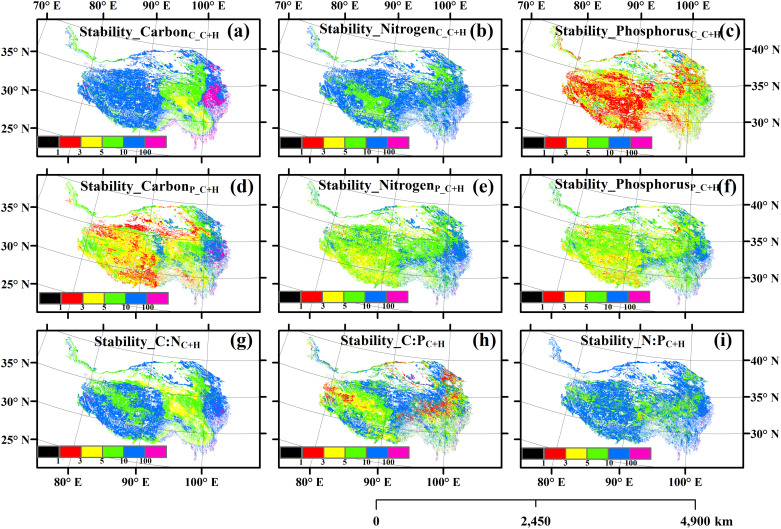
The temporal stability of (a) carbon content, (b) nitrogen content, (c) phosphorus content, (d) carbon pool, (e) nitrogen pool, (f) phosphorus pool, (g) ratio of carbon to nitrogen (C:N), (h) ratio of carbon to phosphorus (C:P), and (i) ratio of nitrogen to phosphorus (N:P). C + H: simultaneously affected by climate change and human activities.

**Fig 3:**
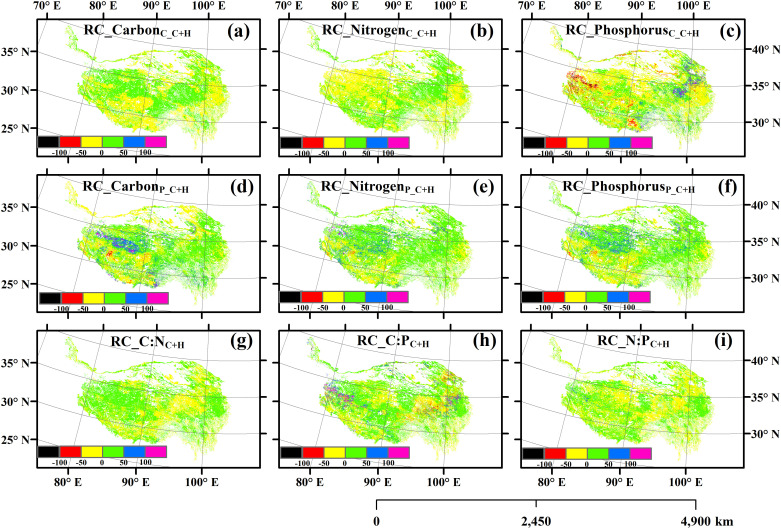
The relative change of (a) carbon content, (b) nitrogen content, (c) phosphorus content, (d) carbon pool, (e) nitrogen pool, (f) phosphorus pool, (g) ratio of carbon to nitrogen (C:N), (h) ratio of carbon to phosphorus (C:P), and (i) ratio of nitrogen to phosphorus (N:P). C + H: simultaneously affected by climate change and human activities.

The spatial average Carbon_C_C+H_, Nitrogen_C_C+H_, Phosphorus_C_C+H_, Carbon_P_C+H_, Nitrogen_P_C+H_, Phosphorus_P_C+H_, C:N_C+H_, C:P_C+H_ and N:P_C+H_ was 29.96% (21.84–41.71%), 1.56% (0.80–2.35%), 0.32% (0.10–1.07%), 28.44 g C m^-2^ (6.35–73.91 g C m^-2^), 0.82 g N m^-2^ (0.27–1.77 g N m^-2^), 0.07 g P m^-2^ (0.02–0.17 g P m^-2^), 30.39 (19.45–51.89), 271.16 (75.36–657.59) and 9.59 (5.72–13.39) across all the grassland regions in 2000–2022, respectively. The spatial average RC_Carbon_C_C+H_, RC_Nitrogen_C_C+H_, RC_Phosphorus_C_C+H_, RC_Carbon_P_C+H_, RC_Nitrogen_P_C+H_, RC_Phosphorus_P_C+H_, RC_C:N_C+H_, RC_C:P_C+H_ and RC_N:P_C+H_ was 0.71% (from –43.29% to 60.87%), 0.28% (from –53.18% to 77.93%), –1.64% (from –372.10% to 572.20%), 10.28% (from –241.45% to 838.13%), 11.05% (from –99.04% to 353.09%), 10.29% (from –116.61% to 361.75%), 2.36% (from –44.45% to 159.80%), 8.46% (from –489.82% to 677.14%) and 0.42% (from –98.78% to 202.33%), respectively.

**Fig 4:**
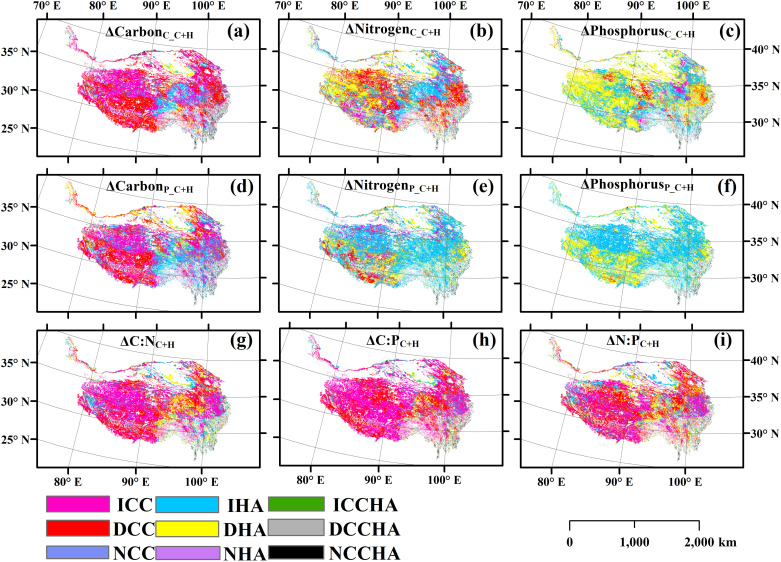
The relative contribution of climate change and human activities to the change of (a) carbon content, (b) nitrogen content, (c) phosphorus content, (d) carbon pool, (e) nitrogen pool, (f) phosphorus pool, (g) ratio of carbon to nitrogen (C:N), (h) ratio of carbon to phosphorus (C:P), and (i) ratio of nitrogen to phosphorus (N:P).

**Fig 5:**
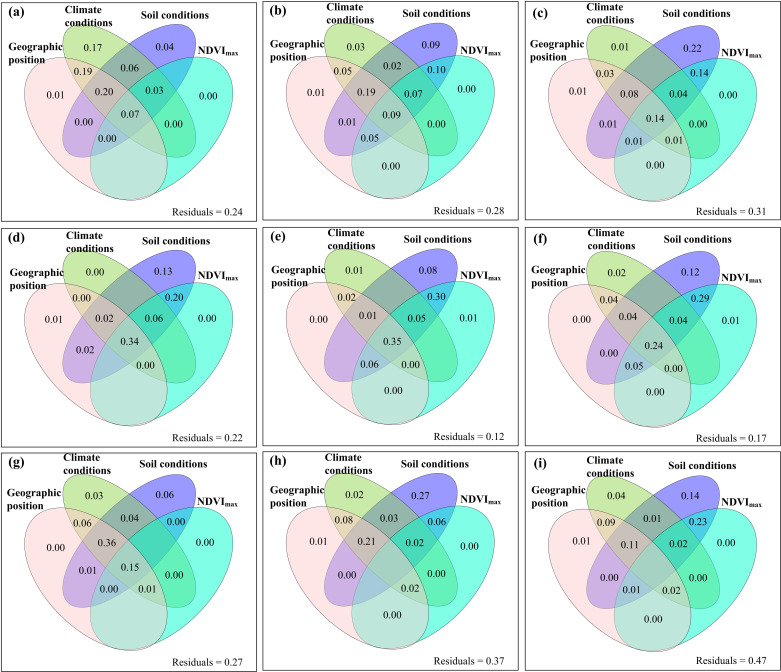
The relative contribution of geographic position, climate conditions (mean annual air temperature, precipitation and radiation), soil conditions (mean soil organic carbon, total nitrogen, total phosphorus, ammonium nitrogen, nitrate nitrogen, available phosphorus, pH, ratio of soil organic carbon to total nitrogen, ratio of soil organic carbon to total phosphorus, and ratio of total nitrogen to total phosphorus) and mean annual maximum normalized difference vegetation index (NDVI_max_) to the mean (a) carbon content, (b) nitrogen content, (c) phosphorus content, (d) carbon pool, (e) nitrogen pool, (f) phosphorus pool, (g) ratio of carbon to nitrogen (C:N), (h) ratio of carbon to phosphorus (C:P), and (i) ratio of nitrogen to phosphorus (N:P) under the combined effects of climate change and human activities.

**Fig 6:**
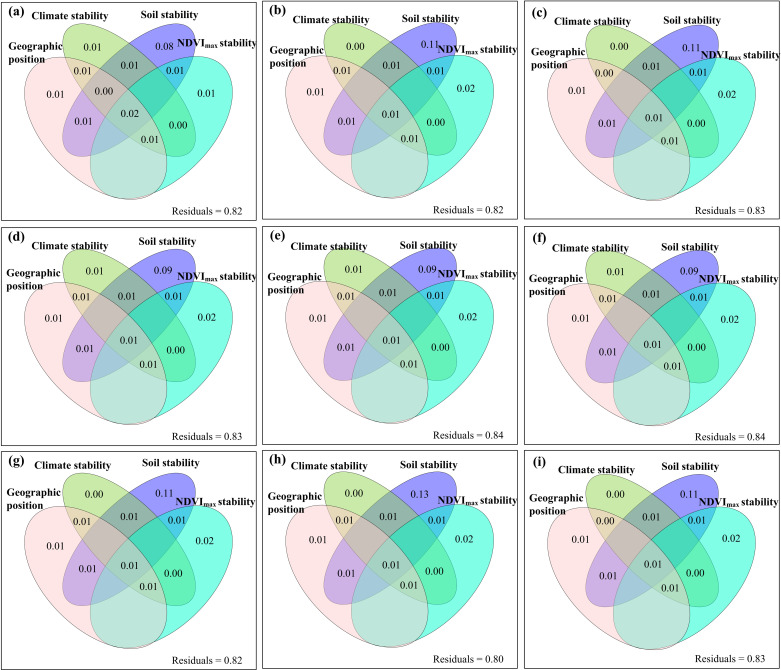
The relative contribution of geographic position, climate stability (the temporal stability of annual air temperature, precipitation and radiation), soil stability (the temporal stability of soil organic carbon, total nitrogen, total phosphorus, ammonium nitrogen, nitrate nitrogen, available phosphorus, pH, ratio of soil organic carbon to total nitrogen, ratio of soil organic carbon to total phosphorus, and ratio of total nitrogen to total phosphorus) and the temporal stability of maximum normalized difference vegetation index (NDVI_max_) to the temporal stability of (a) carbon content, (b) nitrogen content, (c) phosphorus content, (d) carbon pool, (e) nitrogen pool, (f) phosphorus pool, (g) ratio of carbon to nitrogen (C:N), (h) ratio of carbon to phosphorus (C:P), and (i) ratio of nitrogen to phosphorus (N:P) under the combined effects of climate change and human activities.

**Fig 7:**
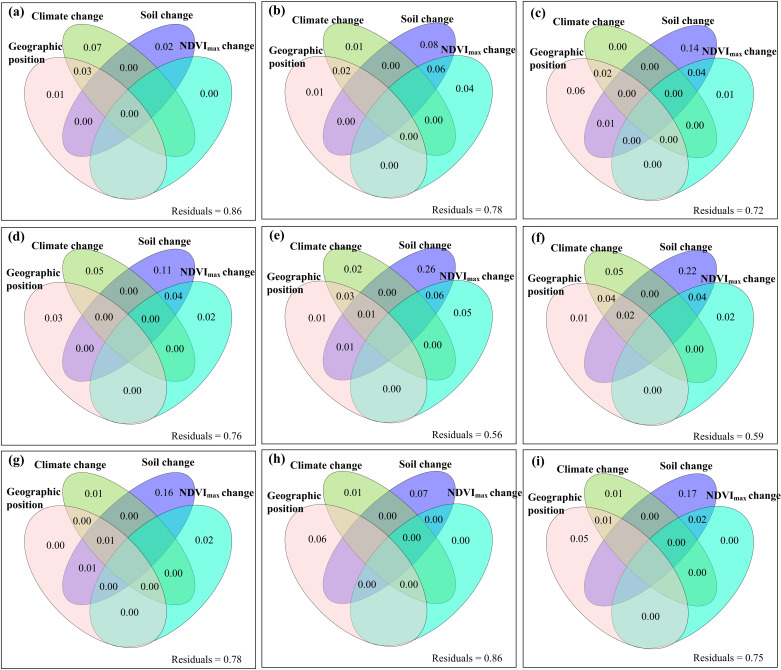
The relative contribution of geographic position, climate change (the change rate of annual air temperature, precipitation and radiation), soil change (the relative change of soil organic carbon, total nitrogen, total phosphorus, ammonium nitrogen, nitrate nitrogen, available phosphorus, pH, ratio of soil organic carbon to total nitrogen, ratio of soil organic carbon to total phosphorus, and ratio of total nitrogen to total phosphorus) and the change rate of maximum normalized difference vegetation index (NDVI_max_) to the relative change of (a) carbon content, (b) nitrogen content, (c) phosphorus content, (d) carbon pool, (e) nitrogen pool, (f) phosphorus pool, (g) ratio of carbon to nitrogen (C:N), (h) ratio of carbon to phosphorus (C:P), and (i) ratio of nitrogen to phosphorus (N:P) under the combined effects of climate change and human activities.

The relative contributions of climate change and human activities to the changes of these variables of the aboveground parts of plants were spatial heterogeneous (Fig 4). The areas where the changes in Nitrogen_C_C+H_, Phosphorus_C_C+H_, Nitrogen_P_C+H_ and Phosphorus_P_C+H_ were attributed to human activities were greater, while the areas where the changes in the other five variables were attributed to climate change were greater (**Fig 4**). The relative changes of these variables of the aboveground parts of plants did not decrease with the increase of their temporal stability (S11–S13 Figs in S1 File). The independent variables of geographical location, climatic factors, soil factors and vegetation factors all had exclusive impacts on the content of carbon, nitrogen and phosphorus of plants themselves, as well as on their temporal stability and relative changes (Figs 5–7, S14–S19 in S1 File).

## 4. Discussion

The aboveground carbon content of plants not only had obvious spatio-temporal variations, but also had an average value of <45% in the alpine grasslands on the Qinghai-Xizang Plateau (Figs 1, S1 in S1 File). This phenomenon was in line with previous studies conducted in alpine grasslands on the Qinghai-Xizang Plateau [26,27]. However, some previous studies multiplied plant biomass directly by 45% in the process of converting it into net primary production (NPP) carbon pool when they examined the accuracy of the NPP models in grasslands on the Qinghai-Xizang Plateau [10,28,29]. Other studies even used 50% as the carbon content of plant biomass [9,30]. Therefore, these previous modeling studies which used fixed and relatively high carbon content, may further cause the risk of overestimating the carbon pools in the alpine grasslands on the Qinghai-Xizang Plateau, especially areas with lower carbon content.

The mean carbon content of the aboveground parts of plants in the alpine grasslands on the Qinghai-Xizang Plateau was < 45%, and the possible mechanisms were as follows. **First**, alpine grasslands plants may possess specific growth strategy and photosynthesis–respiration–balance on the Qinghai-Xizang Plateau. On one hand, in order to adapt to the alpine environments, alpine plants may often give priority to ensuring survival and reproduction rather than carbon accumulation. They may reduce the allocation of biomass for carbon storage. They may curtail the growth of photosynthetic organs (e.g., leaves) and allocate more resources to the growth of roots for absorbing the limited nutrients and water, or synthesize cold-resistant substances (e.g., antifreeze proteins) [31,32]. The synthesis of cold-resistant substances can require the consumption of energy and carbon sources, resulting in a relatively reduced amount of carbon available for accumulation. Meanwhile, plants may increase the synthesis of structural components (e.g., cellulose and lignin in the cell walls) [33,34]. Although these substances contain carbon, they also include other elements, causing the proportion of carbon in the dry matter to relatively decrease. For example, like *Kobresia pygmaea*, it has a relatively developed root system which is used for fixing the plant and absorbing nutrients. On the aboveground part, it reduces the size and number of leaves. Some special antifreeze substances will be synthesized in the leaves, which can make the carbon in the leaves used for synthesizing antifreeze substances instead of being stored, thus reducing the proportion of carbon content in the aboveground part. On the other hand, the low temperature and strong ultraviolet rays in the alpine grasslands can limit the efficiency of photosynthesis [34,35]. The amount of carbon fixed by plants through photosynthesis during the day may be limited [36,37]. However, in the low-temperature environment at night, plants should still need to carry out respiration to maintain basic life activities (e.g., maintaining the ionic balance of cells and material transportation) [38,39]. Due to the fact that the respiration at night can consume a relatively large amount of the carbon accumulated during the day, the net accumulation of carbon can be reduced, and it may be difficult to increase the carbon content in the aboveground parts of plants. **Second**, the seeds of plants in the alpine grasslands on the Qinghai-Xizang Plateau are relatively light in weight, and the growth of their seedlings is slow [40]. Compared with the seeds of plants in low-altitude areas, the seeds of plants in the alpine grasslands on the Qinghai-Xizang Plateau can often accumulate less carbon and other nutrients during their formation due to limiting factors (e.g., low temperature and insufficient nutrient supply in the alpine environment) [41]. Their seeds can be relatively small, with limited carbon reserves available for the initial growth of seedlings. After the seeds germinate, the carbon sources can be easily depleted, making it difficult for the seedlings to quickly establish an efficient photosynthesis system to accumulate carbon. The alpine environment can also cause the initial growth of seedlings to be slow. The low temperature and unstable climatic conditions can require the seedlings to spend more time and energy on adapting to the environment rather than on rapid photosynthesis and carbon accumulation [42]. At this stage, the development of both the roots and leaves of plants can be inhibited. The slow growth of roots may reduce the absorption efficiency of soil nutrients and water, and the slow growth of leaves can make it difficult to rapidly expand the photosynthetic area. For example, the leaves of newly emerged seedlings in the alpine grasslands unfold several weeks slower than those of seedlings in low-altitude areas [43,44]. During this period, the photosynthetic efficiency can be extremely low and the carbon accumulation can even be negligible, which may further affect the overall carbon content of the aboveground parts. **Third**, in the alpine environments on the Qinghai-Xizang Plateau, plants can be faced with interspecific competition and symbiotic relationships with microorganisms (e.g., mycorrhizal fungi) [45], both of which may limit the carbon accumulation of plants to a certain extent. From the perspective of competition among different plant species, in order to obtain limited water and nutrients, plants may change their carbon allocation strategies. In a community composed of various alpine herbaceous plants, when the soil nutrients are scarce in the early stage of growing season, plants (e.g., *Elymus nutans*) can increase the investment in root biomass and correspondingly reduce the carbon allocation for aboveground leaf growth, photosynthesis and carbon accumulation, so as to gain an advantage in the nutrient competition. From the perspective of symbiotic relationships, plants in the alpine environments can rely on mycorrhizal fungi to obtain nutrients, which in turn may lead to an increase in the carbon transported to the mycorrhizal fungi and a decrease in the carbon accumulation of the aboveground parts themselves. For example, *Rhododendron lapponicum* can need to provide about 20–30% of the carbon of its photosynthetic products to mycorrhizal fungi in exchange for nutrients (e.g., phosphorus) [46], thus reducing the carbon content of the aboveground parts. **Fourth**, alpine soils can be characterized by poor nutrient availability and weak microbial activity [14,47]. In some infertile alpine grassland soils, the content of available nitrogen in the soil can be extremely low, resulting in insufficient synthesis of chlorophyll in plants. The leaves can be small in size and light green in color. Under such circumstances, the photosynthesis of plants may be severely restricted, with a small amount of fixed carbon, and the carbon content in the aboveground parts may be correspondingly low. Additionally, in the alpine soil environment of the Plateau, compared with low-altitude areas, the activities of microorganisms involved in the nitrogen cycle (e.g., ammonifying and nitrifying bacteria) can be significantly reduced [48]. This may cause the organic nitrogen in soils to not be converted into ammonium nitrogen and nitrate nitrogen that can be absorbed by plants in a timely manner. Due to the lack of nitrogen, plants can be unable to carry out photosynthesis efficiently [6], and the carbon content in the aboveground parts may be decreased. **Fifth**, the Qinghai-Xizang Plateau not only has a short growth season but also experiences frequent extreme climate events. The growing season of the alpine grasslands on the Qinghai-Xizang Plateau can be relatively short, only lasting 3–4 months, while the growing season of plants in low-altitude areas can reach 6–8 months. During this short growing season, the plants need to complete various life processes (e.g., germination, growth, and reproduction). This may result in relatively less time and resources available for them to accumulate carbon through photosynthesis. Even if the photosynthetic efficiency can be relatively high, the total amount of carbon accumulation and the carbon content in the aboveground parts may be also relatively low, far less than those of the plants in low-altitude areas with a longer growing season. On the other hand, frequent extreme climate events (e.g., frosts, and droughts) can cause damage to plants [49]. Snowstorms may break the aboveground parts of plants and damage the photosynthetic organs. Frosts can damage plant cells, affecting photosynthesis and carbon assimilation. Droughts may cause the stomata of plants to close, reducing the entry of carbon dioxide and thus lowering the efficiency of photosynthesis. All these extreme events can interfere with the normal process of carbon accumulation in plants. For example, in spring, a sudden frost may freeze the newly sprouted buds of plants, damaging their cell structures. These damaged buds cannot carry out photosynthesis normally, and may even die and fall off, interrupting the carbon accumulation in the aboveground parts of plants and reducing the carbon content. **Sixth**, the activities (e.g., excessive grazing and trampling by animals) can inhibit the carbon content in the aboveground parts of plants [50]. The alpine grasslands are the habitats of numerous wild animals (e.g., yaks and Tibetan antelopes). The activities of these animals can affect the aboveground parts of plants and their growth environment, thereby reducing the carbon accumulation of plants. On the one hand, the grazing behavior of animals can directly damage the aboveground parts of plants. Yaks, Tibetan antelopes and other animals can frequently graze on the leaves, stems and other parts of plants, which may reduce the effective area for photosynthesis, resulting in a decrease in the amount of carbon fixed by plants. Moreover, the plants that have been grazed need to consume additional energy and carbon to repair the damaged tissues, leading to a significant reduction in the carbon content of the aboveground parts. On the other hand, taking Tibetan antelopes as an example, when they migrate through alpine grasslands, they leave a large number of hoof prints, which can make the soil compact and deteriorate its aeration and water permeability. This in turn can affect the growth of plant roots and the absorption of nutrients, indirectly reducing the photosynthetic efficiency of plants and causing a decrease in the carbon content.

Neither the individual impacts of climate change and human activities nor their interactive impacts always increased or decreased the contents, pools and ratios of carbon, nitrogen and phosphorus in the aboveground parts of plant community (Fig 3). This finding was in line with previous studies [12,18,51]. These different findings may be due to the following potential causes. **First**, according to theories such as the zonal–vegetation–theory, ecological–gradient–theory and biogeochemical–cycle–theory [52,53], geographical location itself can be important factors in regulating the changes of carbon, nitrogen and phosphorus in the aboveground parts of plants (Fig 7). **Second**, according to the resource–allocation–theory, optimal–partitioning–theory, functional–equilibrium–hypothesis and other such theories [54,55], there may be tradeoff relationships between plant aboveground and root carbon/nitrogen/phosphorus and biomass [56–58], and the trade-off relationships may be dependent on climate and soil conditions. **Third**, according to theories such as the resource–competition–theory [59], both climate change and human activities can alter the competitive relationships among species of plant communities. For example, climate warming may enable some invasive alien plants with originally weaker competitiveness to invade and compete with native plants for resources (e.g., light, water, and nutrients). In terms of carbon, nitrogen, and phosphorus, if the invasive alien plants had stronger nutrient acquisition capabilities, they may seize more nitrogen and phosphorus resources, suppressing the growth and carbon, nitrogen, and phosphorus accumulation in the aboveground parts of native plants. Meanwhile, the different responses of various plants to external disturbances can also lead to dynamic changes in competitive relationships. For instance, when precipitation changes, the competitive advantages of drought-tolerant plants and hygrophilous plants may shift, affecting the overall carbon, nitrogen, and phosphorus accumulation patterns in the aboveground parts of plants in the community. **Fourth,** according to theories such as the ecosystem–resilience–theory and ecosystem–memory–theory [60,61], the impacts of climate change and human activities on the contents of carbon, nitrogen and phosphorus of plants as well as their biomass may all have lagging effects and/or cumulative effects [27,62,63]. Meanwhile, according to theories such as the niche–theory and self–organization–theory [64,65], the carbon, nitrogen, and phosphorus contents and biomass of plants may also have adaptability to long–term external disturbances [66]. For example, epigenetic modifications (e.g., DNA methylation) can regulate gene expression without changing the DNA sequence, enabling plants to respond quickly and produce adaptive changes in the face of environmental changes [67]. Under the influence of long-term climate change or human activities, plants may change the gene expression pattern through epigenetic modifications, affecting physiological processes related to carbon, nitrogen and phosphorus metabolism [68,69]. Long-term grazing pressure may lead to changes in the DNA methylation level in certain gene regions of plants, inhibiting or activating genes related to growth and defense [70,71]. If the expression of genes related to carbon, nitrogen and phosphorus accumulation is inhibited, the carbon, nitrogen and phosphorus accumulation in the aboveground parts of plants will be affected. Moreover, such epigenetic changes may be retained in future generations, affecting the long-term carbon, nitrogen and phosphorus accumulation strategies and adaptability of plants to environmental changes. **Fifth**, according to the intermediate–disturbance–hypothesis, threshold–theory, and other relevant theories [72,73], the influences of climate warming, precipitation change, grazing, nitrogen deposition, etc., on the contents of carbon, nitrogen, and phosphorus of plants as well as their biomass should be all associated with the intensity of the external disturbances to which they were exposed [57,62,74]. For example, if the grazing intensity is moderate, some plants may initiate the compensatory growth mechanism. At this time, plants may increase the photosynthesis rate, absorb more nitrogen from soils to synthesize proteins for building new cells, and absorb more phosphorus involved in energy metabolism and genetic material synthesis. Meanwhile, plants may promote cell division and elongation by regulating the hormonal balance. They will use more assimilated carbon to build new aboveground tissues, increasing the carbon, nitrogen and phosphorus contents in the aboveground parts. Conversely, if the grazing intensity is too high or too low, plants may produce a large amount of abscisic acid, which can prompt premature senescence and abscission of leaves and reduce the area of photosynthesis. Meanwhile, there will be no compensatory carbon, nitrogen and phosphorus accumulation in the aboveground parts of plants. In addition, plants’ responses to environmental changes involve complex gene expression regulatory networks. Under the stress of excessive temperature, the abnormal expression of certain transcription factors may cause plants to reduce the expression of genes related to photosynthesis, decrease carbon assimilation, and simultaneously affect nitrogen metabolism, resulting in a decrease in the carbon, nitrogen, and phosphorus contents in the aboveground parts of plants. **Sixth**, according to theories such as the resource–allocation–theory, phenological–mismatch–theory, ecological–niche–differentiation–theory, and disturbance–theory [75–77], diurnal and seasonal asymmetric climate changes can affect the accumulation of carbon, nitrogen, and phosphorus in the aboveground parts of plants through the re-distribution of resources such as photosynthetic products and the disorders of physiological metabolism and so on [62]. For example, under the condition of seasonally asymmetric climate change, plants may allocate more carbon, nitrogen and phosphorus to the metabolic pathways related to stress resistance or storage organs through hormonal signal regulation to cope with the adverse environment. If this kind of regulation can be not coordinated with the accumulation of carbon, nitrogen and phosphorus, it will affect the normal accumulation of carbon, nitrogen and phosphorus in the above-ground parts. **Seventh**, according to the theory of soil–microbial–community–plant–interaction [78], grazing in different seasons may change the structure and function of soil microbial community [63,79]. During the active growth period of plants (e.g., spring and summer), soil microbial community may adjust due to changes in plant root exudates and litter inputs. The activities of some beneficial microorganisms (e.g., nitrogen-fixing bacteria and phosphorus-solubilizing bacteria) can be enhanced, forming mutually beneficial symbiotic relationships with plants. Microorganisms can help plants obtain more nutrients (e.g., nitrogen and phosphorus), and plants can provide carbon sources and other substances for microorganisms. This interaction can promote plant growth and increase the accumulation of carbon, nitrogen, and phosphorus in the aboveground parts of plants. However, during the non-growing season, soil microbial community may be damaged, affecting nutrient transformation and plant nutrient absorption, and thus affecting the content of carbon, nitrogen, and phosphorus in the aboveground parts of plants. This can reflect the interactive relationships between soil microbial community and plants under different seasonal grazing conditions. **Eighth**, according to theories such as the stoichiometric–balance–theory, nutrient–limitation–theory, plant–soil–feedback–theory and ecosystem–succession–theory [80,81], soil changes caused by climate change and human activities are important driving factors for the changes of carbon, nitrogen and phosphorus in the aboveground parts of plants (Fig 7). For example, after plants absorb nitrogen and phosphorus nutrients, their roots will secrete substances (e.g., organic acids) into soils. These secretions can adjust soil pH and increase the availability of certain nutrients. If the soil nutrients (e.g., phosphorus) are insufficient, the organic acids secreted by the plant roots may increase to dissolve the sparingly soluble phosphorus in soils and improve the effectiveness of phosphorus. Meanwhile, the changes in the carbon, nitrogen, and phosphorus contents in the aboveground parts of plants will affect the quality and quantity of litter. After the litter decomposes, it will return nutrients to soils, thus changing soil nutrient status and pH and in turn feedback to plants. All the situations mentioned above should be spatially heterogeneous. Therefore, they together resulted in the different accumulations of carbon, nitrogen and phosphorus of aboveground plants among different regions.

Typically, we would expect that with the increase in temporal stability, the relative changes of the carbon, nitrogen and phosphorus of the aboveground parts of plants would decrease. This can be because the enhancement of temporal stability may be often accompanied by relatively stable environmental conditions. Specifically, in regions where the climate can be relatively stable, the soil fertility does not change much, and there are no frequent disturbances, the aboveground parts of plants can grow under relatively stable external environmental conditions. In such stable environments, plants can gradually adapt, and the stable temporal conditions are conducive to plants establishing stable growth patterns. For example, under stable light, temperature, and carbon dioxide concentration conditions, the photosynthesis and respiration of plants can tend to reach a relative balance. Taking carbon content as an example, when the balance between photosynthesis and respiration is stable, the range of changes in the carbon content of the aboveground parts of plants can be relatively narrowed. Such a stable environment and balanced physiological processes may limit the variation range of each variable in the aboveground parts of plants, showing a trend of decreasing relative changes with the increase in temporal stability. However, this study found that there were no obvious relationships between the temporal stability of plant carbon, nitrogen and phosphorus and their changes (S11–S13 Figs in S1 File), which was similar with some previous studies [14,15]. This finding indicated that there were no simple negative correlations between the changes of carbon, nitrogen and phosphorus of the aboveground parts of plants and their temporal stability. The dynamic changes of these variables in the aboveground parts of plants can be rather complex. This may be because, in addition to temporal stability, many other factors can have impacts on the carbon, nitrogen and phosphorus of the aboveground parts of plants. The changes of these external factors may prevent the relative changes of carbon, nitrogen and phosphorus of the aboveground parts of plants from decreasing as expected when the temporal stability increased. Specifically, **first**, climate change can have impacts on them. Even if the temporal stability increased, long-term climate change can still lead to significant changes of these variables. For example, warming can accelerate the growth rate of plants and change their phenological periods, causing significant changes in carbon content. Meanwhile, extreme climate events can break the original balance. Even when the overall temporal stability seemed relatively high, occasional extreme climate events can still cause significant fluctuations in these variables. **Second**, soil factors cannot be ignored. The availability of soil nutrients, soil texture and other factors can affect the variables of the aboveground parts of plants [6]. For example, when the contents of nitrogen, phosphorus and potassium in the soil changed suddenly due to changes in soil microbial activities, etc., the ability of plants to absorb nutrients would change, thereby affecting the growth of the aboveground parts and related variables. **Third**, the competitive relationships among plants play obvious roles [82,83]. Within a relatively stable time period, the invasion of new plant species or the change in the competitive advantage of existing plant species can all cause changes in these variables. For example, a highly competitive plant that can gain dominance in the community would compete for more sunlight, water and nutrients, suppressing the growth of the aboveground parts of other plants and increasing the relative changes of these variables. **Fourth**, the symbiotic or interactive relationships between plants and other organisms can also have a potential impact [84]. If the symbiotic relationship between plants and mycorrhizal fungi was damaged due to climate change, etc., the efficiency of plants in absorbing nutrients may decline, resulting in changes in these variables. Changes in the grazing pressure of herbivores can also affect the growth of the aboveground parts of plants. Even if the temporal stability was relatively high, if the number of herbivores suddenly increased, these variables would also change accordingly. **Fifth**, the growth plasticity of plants played a crucial role [85]. Some plants can have high growth plasticity. When the external temporal stability increased, they may adjust the growth of their aboveground parts according to their internal biological clocks or growth strategies. For example, some plants may allocate resources to the development of their aboveground reproductive organs at specific time points, causing significant relative changes in these variables. **Sixth**, the physiological adaptation mechanisms of plants can also have an impact [33,34]. When the environmental conditions were relatively stable within a certain period of time, plants would activate their feedback regulation systems. For example, when plants adapted to the intensity of light, if they were in a stable light environment for a long time, they would adjust the morphology, thickness and pigment content of their leaves to optimize the efficiency of photosynthesis [33]. This physiological regulation process would cause changes in these variables, and the changes did not necessarily decrease as the temporal stability increased, because plants were constantly adapting and optimizing their growth states. **Seventh**, on the one hand, although the overall temporal stability may be relatively high, the differences between regions (e.g., different altitudes) can cause complex and diverse patterns of changes in these variables. For example, in a large mountain range area, even if the overall climate temporal stability increased, the growth conditions and variable changes of the aboveground parts of plants at the high- and low-altitude areas would be different due to the differences in temperature, precipitation and other gradients [86]. On the other hand, the temporal stability was easily disturbed by local environmental factors (e.g., microclimates, etc.), causing relatively large changes in these variables. For example, in the same grassland, water may accumulate in low-lying areas, affecting the growth of plants, while the growth conditions of plants at higher areas were different. This spatial difference may cause these variables to still have relatively large relative changes when the temporal stability increased.

Climate change and human activities homogenized the spatial patterns of the nitrogen–phosphorus pools and the nitrogen–phosphorus contents in the aboveground parts of plants, respectively (S8–S9 Figs in S1 File), which was similar with some previous studies [15]. This phenomenon indicated that under external disturbances, the original spatial heterogeneity of nitrogen and phosphorus of the aboveground parts of plants in the Qinghai-Xizang Plateau may be difficult to maintain. This change had far–reaching impacts, especially on the plant–based food chain, because nitrogen and phosphorus were the key elements in the energy flow and material cycle of the ecosystem. For example, herbivores depended on nitrogen and phosphorus of plants. When the spatial patterns of nitrogen and phosphorus changed, a series of chain reactions would be triggered. This change can affect the food quality and distribution of herbivores. Due to the homogenization of food resources, animals may reduce their exploratory behaviors, and those animals that relied on plants with specific nitrogen and phosphorus contents would even decrease in number because of the changes in food. The changes in the distribution and number of herbivores would further influence the structure of the alpine grasslands. This series of impacts highlighted the importance of the change in the spatial patterns of nitrogen and phosphorus in the ecosystem and also reflected the delicacy of the ecological balance in this region. In addition, homogenization had the following impacts. Reduced the differences in the utilization of nitrogen and phosphorus resources among plants in different spatial positions. Some plant species that adapted to specific nitrogen–phosphorus spatial patterns may decrease due to the loss of their advantages. Reduced the complexity and diversity of the nitrogen–phosphorus cycling in the alpine grasslands. In the original highly heterogeneous ecosystems, there were multiple nitrogen–phosphorus transformation and storage methods, which may be simplified due to homogenization, resulting in the decline of the ecosystem’s buffering and regulating abilities of nitrogen and phosphorus. Made the ecosystem structure single and more vulnerable to the impact of external disturbances. When facing pest and disease infestations or extreme climate events, the homogenized ecosystems may lack spatial differences to buffer these disturbances, affecting functions and weakening the recovery ability. Caused the convergence of ecological characteristics in different regions. The original characteristic spatial patterns of plant nitrogen and phosphorus in different valleys and plateau surfaces on the Qinghai–Xizang Plateau may disappear, and the ecological landscapes and processes may become similar. This may not only reduce the regional ecological uniqueness, affect economic activities, but also bring challenges to the protection and management of the ecosystem, and the original protection strategies may no longer be applicable.

## 5. Conclusions

The aboveground carbon content of plants ranged from 21.84% to 41.71%, which was obviously <45%. This warned that when constructing ecological models in the future, the plant carbon content cannot be simply set as a fixed value. The actual range should be taken into account. Because if the model uses inaccurate fixed parameters, it will lead to incorrect estimations of processes such as plant carbon absorption and carbon storage, and further affect the simulation accuracy of the carbon cycle. The total amount of the aboveground carbon pool of plants in the alpine grasslands on the Qinghai-Xizang Plateau was 0.04 Pg C in 2022. Except that the spatial average of the change in phosphorus content decreased by 1.64%, all the other variables increased by 0.28–11.05%, showing current climate change and human activities can be conducive to the carbon and nitrogen sequestration of the aboveground parts of plants. Climate change and human activities can lead to the spatial homogenization of nitrogen and phosphorus in the aboveground parts of plants, which may reduce the diversity, complexity and stability of ecosystems, and even change regional ecological characteristics, thereby bringing about a series of challenges. The change of plant carbon, nitrogen, and phosphorus were not related to their temporal stabilities. Therefore, when constructing ecological system models, one cannot simply assume that there exists a certain fixed relationship between the temporal changes and temporal stabilities of plant carbon, nitrogen, and phosphorus. For ecological system managers, they also cannot easily judge the health status or stability of the ecological system merely based on the change situation of a certain element among plant carbon, nitrogen, and phosphorus.

## Supporting information

S1 File **S1 Fig**. Observed carbon content of plant aboveground parts under the sole effect of climate change or the combined effects of climate change and human activities. **S2 Fig**. The spatial distribution of (a) carbon content, (b) nitrogen content, (c) phosphorus content, (d) carbon pool, (e) nitrogen pool, (f) phosphorus pool, (g) ratio of carbon to nitrogen (C:N), (h) ratio of carbon to phosphorus (C:P), and (i) ratio of nitrogen to phosphorus (N:P). C: solely affected by climate change. **S3 Fig**. The spatial distribution of (a) carbon content, (b) nitrogen content, (c) phosphorus content, (d) carbon pool, (e) nitrogen pool, (f) phosphorus pool, (g) ratio of carbon to nitrogen (C:N), (h) ratio of carbon to phosphorus (C:P), and (i) ratio of nitrogen to phosphorus (N:P). H: solely affected by human activities. **S4 Fig**. The temporal stability of (a) carbon content, (b) nitrogen content, (c) phosphorus content, (d) carbon pool, (e) nitrogen pool, (f) phosphorus pool, (g) ratio of carbon to nitrogen (C:N), (h) ratio of carbon to phosphorus (C:P), and (i) ratio of nitrogen to phosphorus (N:P). C: solely affected by climate change. **S5 Fig**. The temporal stability of (a) carbon content, (b) nitrogen content, (c) phosphorus content, (d) carbon pool, (e) nitrogen pool, (f) phosphorus pool, (g) ratio of carbon to nitrogen (C:N), (h) ratio of carbon to phosphorus (C:P), and (i) ratio of nitrogen to phosphorus (N:P). H: solely affected by human activities. **S6 Fig**. The relative change of (a) carbon content, (b) nitrogen content, (c) phosphorus content, (d) carbon pool, (e) nitrogen pool, (f) phosphorus pool, (g) ratio of carbon to nitrogen (C:N), (h) ratio of carbo n to phosphorus (C:P), and (i) ratio of nitrogen to phosphorus (N:P). C: solely affected by climate change. **S7 Fig**. The relative change of (a) carbon content, (b) nitrogen content, (c) phosphorus content, (d) carbon pool, (e) nitrogen pool, (f) phosphorus pool, (g) ratio of carbon to nitrogen (C:N), (h) ratio of carbon to phosphorus (C:P), and (i) ratio of nitrogen to phosphorus (N:P). H: solely affected by human activities. **S8 Fig**. Relationships between (a) the change rate of carbon content and carbon content, (b) the change rate of nitrogen content and nitrogen content, (c) the change rate of phosphorus content and phosphorus content, (d) the change rate of carbon pool and carbon pool, (e) the change rate of nitrogen pool and nitrogen pool, (f) the change rate of phosphorous pool and phosphorus pool, (g) the change rate of the ratio of carbon to nitrogen (C:N) and the C:N, (h) the change rate of the ratio of carbon to phosphorus (C:P) and the C:P, and (i) the change rate of the ratio of nitrogen to phosphorus (N:P) and the N:P. C: solely affected by climate change. **S9 Fig**. Relationships between (a) the change rate of carbon content and carbon content, (b) the change rate of nitrogen content and nitrogen content, (c) the change rate of phosphorus content and phosphorus content, (d) the change rate of carbon pool and carbon pool, (e) the change rate of nitrogen pool and nitrogen pool, (f) the change rate of phosphorous pool and phosphorus pool, (g) the change rate of the ratio of carbon to nitrogen (C:N) and the C:N, (h) the change rate of the ratio of carbon to phosphorus (C:P) and the C:P, and (i) the change rate of the ratio of nitrogen to phosphorus (N:P) and the N:P. H: solely affected by human activities. **S10 Fig.** Relationships between (a) the change rate of carbon content and carbon content, (b) the change rate of nitrogen content and nitrogen content, (c) the change rate of phosphorus content and phosphorus content, (d) the change rate of carbon pool and carbon pool, (e) the change rate of nitrogen pool and nitrogen pool, (f) the change rate of phosphorous pool and phosphorus pool, (g) the change rate of the ratio of carbon to nitrogen (C:N) and the C:N, (h) the change rate of the ratio of carbon to phosphorus (C:P) and the C:P, and (i) the change rate of the ratio of nitrogen to phosphorus (N:P) and the N:P. C + H: simultaneously affected by climate change and human activities. **S11 Fig**. Relationships between (a) the relative change of carbon content and the temporal stability of carbon content, (b) the relative change of nitrogen content and the temporal stability of nitrogen content, (c) the relative change of phosphorus content and the temporal stability of phosphorus content, (d) the relative change of carbon pool and the temporal stability of carbon pool, (e) the relative change of nitrogen pool and the temporal stability of nitrogen pool, (f) the relative change of phosphorous pool and the temporal stability of phosphorus pool, (g) the relative change of the ratio of carbon to nitrogen (C:N) and the temporal stability of the C:N, (h) the relative change of the ratio of carbon to phosphorus (C:P) and the temporal stability of the C:P, and (i) the relative change of the ratio of nitrogen to phosphorus (N:P) and the temporal stability of the N:P. C + H: simultaneously affected by climate change and human activities. S12 Fig. Relationships between (a) the relative change of carbon content and the temporal stability of carbon content, (b) the relative change of nitrogen content and the temporal stability of nitrogen content, (c) the relative change of phosphorus content and the temporal stability of phosphorus content, (d) the relative change of carbon pool and the temporal stability of carbon pool, (e) the relative change of nitrogen pool and the temporal stability of nitrogen pool, (f) the relative change of phosphorous pool and the temporal stability of phosphorus pool, (g) the relative change of the ratio of carbon to nitrogen (C:N) and the temporal stability of the C:N, (h) the relative change of the ratio of carbon to phosphorus (C:P) and the temporal stability of the C:P, and (i) the relative change of the ratio of nitrogen to phosphorus (N:P) and the temporal stability of the N:P. C: solely affected by climate change. **S13 Fig**. Relationships between (a) the relative change of carbon content and the temporal stability of carbon content, (b) the relative change of nitrogen content and the temporal stability of nitrogen content, (c) the relative change of phosphorus content and the temporal stability of phosphorus content, (d) the relative change of carbon pool and the temporal stability of carbon pool, (e) the relative change of nitrogen pool and the temporal stability of nitrogen pool, (f) the relative change of phosphorous pool and the temporal stability of phosphorus pool, (g) the relative change of the ratio of carbon to nitrogen (C:N) and the temporal stability of the C:N, (h) the relative change of the ratio of carbon to phosphorus (C:P) and the temporal stability of the C:P, and (i) the relative change of the ratio of nitrogen to phosphorus (N:P) and the temporal stability of the N:P. H: solely affected by human activities. **S14 Fig**. The relative contribution of geographic position, climate conditions (mean annual air temperature, precipitation and radiation), soil nutrient conditions (mean soil organic carbon, total nitrogen, total phosphorus, ammonium nitrogen, nitrate nitrogen, available phosphorus, ratio of soil organic carbon to total nitrogen, ratio of soil organic carbon to total phosphorus, and ratio of total nitrogen to total phosphorus) and mean soil pH to the mean (a) carbon content, (b) nitrogen content, (c) phosphorus content, (d) carbon pool, (e) nitrogen pool, (f) phosphorus pool, (g) ratio of carbon to nitrogen (C:N), (h) ratio of carbon to phosphorus (C:P), and (i) ratio of nitrogen to phosphorus (N:P) under the single effect of climate change. **S15 Fig**. The relative contribution of geographic position, climate conditions (mean annual air temperature, precipitation and radiation), soil conditions (mean soil organic carbon, total nitrogen, total phosphorus, ammonium nitrogen, nitrate nitrogen, available phosphorus, pH, ratio of soil organic carbon to total nitrogen, ratio of soil organic carbon to total phosphorus, and ratio of total nitrogen to total phosphorus) and mean annual maximum normalized difference vegetation index (NDVImax) to the mean (a) carbon content, (b) nitrogen content, (c) phosphorus content, (d) carbon pool, (e) nitrogen pool, (f) phosphorus pool, (g) ratio of carbon to nitrogen (C:N), (h) ratio of carbon to phosphorus (C:P), and (i) ratio of nitrogen to phosphorus (N:P) under the single effect of human activities. **S16 Fig**. The relative contribution of geographic position, climate stability (the temporal stability of annual air temperature, precipitation and radiation), soil nutrient. en to total phosphorus) and the temporal stability of soil pH to the temporal stability of (a) carbon content, (b) nitrogen content, (c) phosphorus content, (d) carbon pool, (e) nitrogen pool, (f) phosphorus pool, (g) ratio of carbon to nitrogen (C:N), (h) ratio of carbon to phosphorus (C:P), and (i) ratio of nitrogen to phosphorus (N:P) under the single effect of climate change. **S17 Fig**. The relative contribution of geographic position, climate stability (the temporal stability of annual air temperature, precipitation and radiation), soil stability (the temporal stability of soil organic carbon, total nitrogen, total phosphorus, ammonium nitrogen, nitrate nitrogen, available phosphorus, pH, ratio of soil organic carbon to total nitrogen, ratio of soil organic carbon to total phosphorus, and ratio of total nitrogen to total phosphorus) and the temporal stability of maximum normalized difference vegetation index (NDVImax) to the temporal stability of (a) carbon content, (b) nitrogen content, (c) phosphorus content, (d) carbon pool, (e) nitrogen pool, (f) phosphorus pool, (g) ratio of carbon to nitrogen (C:N), (h) ratio of carbon to phosphorus (C:P), and (i) ratio of nitrogen to phosphorus (N:P) under the single effect of human activities. **S18 Fig**. The relative contribution of geographic position, climate change (the change rate of annual air temperature, precipitation and radiation), soil nutrient change (the relative change of soil organic carbon, total nitrogen, total phosphorus, ammonium nitrogen, nitrate nitrogen, available phosphorus, ratio of soil organic carbon to total nitrogen, ratio of soil organic carbon to total phosphorus, and ratio of total nitrogen to total phosphorus) and the relative change of soil pH to the relative change of (a) carbon content, (b) nitrogen content, (c) phosphorus content, (d) carbon pool, (e) nitrogen pool, (f) phosphorus pool, (g) ratio of carbon to nitrogen (C:N), (h) ratio of carbon to phosphorus (C:P), and (i) ratio of nitrogen to phosphorus (N:P) under the single effect of climate change. S19 Fig. The relative contribution of geographic position, climate change (the change rate of annual air temperature, precipitation and radiation), soil change (the relative change of soil organic carbon, total nitrogen, total phosphorus, ammonium nitrogen, nitrate nitrogen, available phosphorus, pH, ratio of soil organic carbon to total nitrogen, ratio of soil organic carbon to total phosphorus, and ratio of total nitrogen to total phosphorus) and the change rate of maximum normalized difference vegetation index (NDVImax) to the relative change of (a) carbon content, (b) nitrogen content, (c) phosphorus content, (d) carbon pool, (e) nitrogen pool, (f) phosphorus pool, (g) ratio of carbon to nitrogen (C:N), (h) ratio of carbon to phosphorus (C:P), and (i) ratio of nitrogen to phosphorus (N:P) under the single effect of human activities.
(RAR)

S2 File Minimal data set1. (RAR)

S3 File Minimal data set2. (RAR)
